# A Novel Sensor Selection and Power Allocation Algorithm for Multiple-Target Tracking in an LPI Radar Network

**DOI:** 10.3390/s16122193

**Published:** 2016-12-21

**Authors:** Ji She, Fei Wang, Jianjiang Zhou

**Affiliations:** Key Laboratory of Radar Imaging and Microwave Photonics, Ministry of Education, Nanjing University of Aeronautics and Astronautics, Nanjing 211106, China; designnuaa@sina.com (J.S.); wangxiaoxian@nuaa.edu.cn (F.W.)

**Keywords:** low probability of intercept (LPI), mutual information (MI), multiple-target tracking, radar network

## Abstract

Radar networks are proven to have numerous advantages over traditional monostatic and bistatic radar. With recent developments, radar networks have become an attractive platform due to their low probability of intercept (LPI) performance for target tracking. In this paper, a joint sensor selection and power allocation algorithm for multiple-target tracking in a radar network based on LPI is proposed. It is found that this algorithm can minimize the total transmitted power of a radar network on the basis of a predetermined mutual information (MI) threshold between the target impulse response and the reflected signal. The MI is required by the radar network system to estimate target parameters, and it can be calculated predictively with the estimation of target state. The optimization problem of sensor selection and power allocation, which contains two variables, is non-convex and it can be solved by separating power allocation problem from sensor selection problem. To be specific, the optimization problem of power allocation can be solved by using the bisection method for each sensor selection scheme. Also, the optimization problem of sensor selection can be solved by a lower complexity algorithm based on the allocated powers. According to the simulation results, it can be found that the proposed algorithm can effectively reduce the total transmitted power of a radar network, which can be conducive to improving LPI performance.

## 1. Introduction

In recent decades, LPI radar networks have received great attention from academic researchers and radar engineers [1,2]. Compared with traditional monostatic and bistatic radar, a radar network system presents a great number of advantages such as improved detection and tracking performance, more flexible system arrangement, as well as better information retrieval capability [3,4]. As a result, power allocation and sensor selection in radar networks are receiving more attention [5,6].

In modern electronic warfare, in order to successfully detect and track targets, radar signal processing systems must first suppress interference from enemy systems at the same angle and Doppler as that of each potential target, such as a jammer whose interference is typically modeled as structured [7,8,9]. Also, radar has to deal with many advanced threats such as electronic warfare support (ES), radar warning receivers (RWRs), and anti-radiation missiles (ARMs). Consequently, the notion of LPI design is an indispensable and vital tool for the military [10,11]. In addition to LPI radar design, it is essential to maintain secure communication with low probability of intercept from hostile interceptors. The approach of embedding sensitive information into radar emissions by changing the waveform during each radar pulse has been researched in much of the literature [12,13,14].

Recently, the study of LPI optimization for target detection and tracking concerning radar network systems has received increasing impetus. Shi et al. [15,16] proposed two novel LPI optimization schemes for radar network systems in a single-target scenario based on mutual information (MI) and minimum mean square error (MMSE). Narykov et al. [17,18] investigated a sensor selection algorithm for target tracking by using multiple phase array radars, which aims at adaptively selecting the sensor and its parameters, optimizing the resource loading, and guaranteeing a certain level of tracking performance. 

Almost all of those studies focus on a single-target scenario, which may not be valid for multiple-target scenarios. References [19,20,21,22] are the representative works published in the subject of optimization problem concerning sensor selection and power allocation in multiple-target scenarios. Chavali et al. [19] employed a cognitive radar network for multiple-target tracking and proposed an optimization criterion for the antenna selection and power allocation based on the minimization of the Posterior Cramer-Rao bound in a complex urban environment. Godrich et al. [20] proposed a cluster resource scheme for tracking the location of multiple-target with radar network system. In terms of LPI radar networks for multiple-target scenarios, Andargoli et al. [21] proposed a target assignment and power allocation algorithm in search tasks for LPI design by assuming that only a single radar is assigned to each target. Xie et al. [22] proposed a joint selection and power allocation (JSPA) strategy for multiple-target tracking in the decentralized radar network to support the resource aware design. However, there are some limitations of these studies mentioned above. Reference [19] aims at maximizing the achievable tracking accuracy under the conditions of a given power budget which cannot reduce the intercept probability of radar network. In reference [20], a certain localization accuracy threshold may be acquired based on using a smallest subset of the available sensors, while the transmitting power of each selected sensor is not optimized. The target assignment and power allocation strategy analyzed in reference [21] is just suitable for search radar network with ESM support. In reference [22], the worst case tracking Posterior Cramer-Rao Lower Bound (PCRLB) is utilized as a metric for JSPA strategy, and it cannot improve the low probability of intercept performance of radar network.

To conclude, the problem of sensor selection and power allocation to realize LPI optimization of radar network system in multiple-target scenarios, which has never been taken into consideration, needs to be analyzed in detail. 

The remainder of this paper is organized as follows. Section 2 presents the radar network sensitivity and signal model. Section 3 mainly introduces the MI of radar network between the target impulse response and the reflected signal. A sensor selection and power allocation algorithm in an LPI radar network based on the predefined MI threshold has been proposed in Section 4. The resulting non-convex LPI optimization problem, which contains two variables, can be reformulated as a power allocation problem and a sensor selection problem. The power allocation problem is solved in Section 4.1, while the sensor selection problem is solved in Section 4.2. In Section 4.3, K-Nearest Neighbor (KNN) algorithm [23] is adopted to solve the problem of data association and sequential importance resampling particle filter (SIR-PF) technique [24] is adopted to achieve the target state estimation. Then, numerical examples are provided in Section 5. Finally, concluding remarks are given in Section 6.

## 2. Radar Network System Model

### 2.1. Sensitivity of the Radar Network System

A two-dimensional radar network system with N monostatic radars where each transmitter is collocated with a single receiver can be considered, which share data and information to improve overall performance. A set of Q extended targets in the two-dimensional plane where the radar system is located is assumed to be detected and tracked. All radars in the radar network are synchronized properly. The time synchronization of the radar network is achieved by relying on the global position system (GPS) [25]. That is, each transmitter and each receiver in the radar network synchronizes to an accurate clock which is calibrated by GPS. In this paper, it is assumed that transmitters in the radar network have a certain beam-forming capability and each target tracking uses a selected transmitter to transmit signals. The radar network works cooperatively such that the selected active radar transmit waveforms to the corresponding target and all radars can receive and process these echoes that are reflected from the targets. One radar node of the radar network is set as fusion center, in which information fusion, sensor selection and power allocation have been given.

Each target tracking is performed using one transmitter and N receivers of the radar network. It can be seen that the whole network can be broken down into 1×N transmitter-receiver pairs to track each target, each of which has a bistatic component contributing to the overall signal-to-noise ratio (SNR). The SNR of each transmitter-receiver pair can be achieved by the bistatic radar equation. The overall SNR of radar network for target q can be calculated as the sum of the partial SNR of each transmitter-receiver pair [26], as follows:

(1) $${\mathrm{SNR}}_{q}=\sum_{i=1}^{N}\sum_{j=1}^{N}\frac{u_{i}^{q}E_{ti}G_{ti}G_{rj}\sigma_{ij}^{q}\lambda_{i}^{2}}{{\left(4\pi \right)}^{3}kT_{s}B_{i}L_{ij}N_{Fj}{R_{ti}^{q}}^{2}{R_{rj}^{q}}^{2}}$$

where $E_{ti}$ is the transmitted power of radar i; $G_{ti}$ is the transmit antenna gain of radar i; $G_{rj}$ is the receive antenna gain of radar j; $\sigma_{ij}^{q}$ is the radar cross section (RCS) of target *q* for radar i and radar j; $\lambda_{i}$ is the transmitted wavelength of radar i; k is Boltzmann’s constant; $T_{s}$ is the receiving system noise temperature; $B_{i}$ is the bandwidth of the matched filter for the transmitted waveform of radar i; $L_{ij}$ is the system loss for radar i and radar j; $N_{Fj}$ is the noise factor at radar j; $R_{ti}^{q}$ is the distance from radar i to target q; $R_{rj}^{q}$ is the distance from target q to radar j. Sensor selection index. $u_{i}^{q}$ is a binary variable can be denoted as:

(2) $$u_{i}^{q}=\{\begin{array}{l} 1\text{ if radar }i\text{ is selected to track target }q\\ 0\text{ otherwise} \end{array}$$

It should satisfy $\sum_{i=1}^{N}u_{i}^{q}=1$ due to the assumption that only one radar node can be selected to track target q in an active way at each time instant.

### 2.2. Radar Network Signal Model

The transmitted signal of radar *i* when it is assigned to target *q* at time instant *k* can be denoted as $s_{i}^{q}(k)$. Then the received signal of radar *j* from target *q* at time instant *k* can be described as:

(3) $$y_{j}^{q}(k)=s_{i}^{q}(k)h_{ij}^{q}+w_{j}(k)$$

where $h_{ij}^{q}$ is the path gain from radar *i* to radar *j* for target *q*; *w_j_*(*k*) refers to the noise in radar *j*. Assuming that the number of samples within the duration of transmitted waveform is *K*, *K* > *N*, then the received signals of radar *j* can be expressed as:

(4) $$y_{j}^{q}=s_{i}^{q}h_{ij}^{q}+w_{j}$$

where $y_{j}^{q}={\left[y_{j}^{q}(1)y_{j}^{q}(2)...y_{j}^{q}(K)\right]}^{T}\in {\mathbb{C}}^{K\times 1}$; $s_{i}^{q}={\left[s_{i}^{q}(1)s_{i}^{q}(2)...s_{i}^{q}(K)\right]}^{T}\in {\mathbb{C}}^{K\times 1}$; $w_{j}={\left[w_{j}(1)w_{j}(2)...w_{j}(K)\right]}^{T}\in {\mathbb{C}}^{K\times 1}$. When defining **U***^q^* as an N×N diagonal matrix that has sensor selection index $u_{i}^{q}$ as its diagonal entries, then the received signal matrix for target q $Y^{q}=\left[y_{1}^{q},y_{2}^{q},...,y_{N}^{q}\right]\in {\mathbb{C}}^{K\times N}$ is given by:

(5) $$Y^{q}=S^{q}U^{q}H^{q}+W$$

where $H^{q}=\left[h_{1}^{q},h_{2}^{q},...,h_{N}^{q}\right]\in {\mathbb{C}}^{N\times N}$, $h_{j}^{q}={\left[h_{1j}^{q}h_{2j}^{q}...h_{Nj}^{q}\right]}^{T}\in {\mathbb{C}}^{N\times 1}$ is the path gain vector for radar *j*; $S^{q}=\left[s_{1}^{q},s_{2}^{q},...,s_{N}^{q}\right]\in {\mathbb{C}}^{K\times N}$ and $W=\left[w_{1},w_{2},...,w_{N}\right]\in {\mathbb{C}}^{K\times N}$.

To facilitate our ensuing analysis, Equation (5) involves the following assumptions.

(1) All radars in the radar network are sufficiently separated, and the transmitter–target–receiver geometries are different due to propagation distances and antenna gains. Based on the discussions in reference [27], the path gain $h_{ij}^{q}$ includes two parts, namely the target reflection coefficient $g_{ij}^{q}$ and the propagation loss factor $p_{ij}^{q}$.
(2) The targets are comprised of a large number of small independent and identically distributed (i.i.d.) random scatterers, then $g_{j}^{q}∼CN(0,\sigma_{g}^{2}I_{N})$ can be got based on the central limit theorem [28], where $g_{j}^{q}={\left[g_{1j}^{q}g_{2j}^{q}...g_{Nj}^{q}\right]}^{T}\in {\mathbb{C}}^{N\times 1}$. The propagation loss $p_{ij}^{q}$ is concerned with target proximity and antenna properties:
  (6) $$p_{ij}^{q}=\frac{\kappa }{R_{ti}^{q}R_{rj}^{q}\sqrt{G_{ti}G_{rj}}}$$
  where κ is a constant. The $p_{ij}^{q}$’s would differ from one another which can be easily obtained if transmitters have a certain beam forming capability and they track targets cooperatively assuming beam synchronization.
(3) All receivers are homogeneous and the receiver noises are white Gaussion noise, so those of the $w_{j}$ are i.i.d. complex Gaussion vectors with distribution $w_{j}∼CN(0,\sigma_{w}^{2}I_{K})$.
(4) $H^{q}$ and W are mutually independent.

According to these assumptions, Equation (5) can be rewritten as:

(7) $$Y^{q}=S^{q}U^{q}(G^{q}⊙P^{q})+W$$

where the target scatterer matrix $G^{q}=\left[g_{1}^{q},g_{2}^{q},...,g_{N}^{q}\right]\in {\mathbb{C}}^{N\times N}$; the propagation loss matrix $P^{q}=\left[p_{1}^{q},p_{2}^{q},...,p_{N}^{q}\right]\in {\mathbb{C}}^{N\times N}$, $p_{j}^{q}={\left[p_{1j}^{q}p_{2j}^{q}...p_{Nj}^{q}\right]}^{T}\in {\mathbb{C}}^{N\times 1}$; ⊙ indicates Hadamard product.

## 3. Mutual Information

The notion of mutual information has been applied for radar networks to measure the capability of estimating target parameters. The work presented in reference [29] provides the MI based criterion view in designing radar waveform. It is shown that a larger MI means a better ability to estimate target parameters but does not guarantee an optimal LPI performance. Our main goal is to optimize the LPI performance by reducing the total transmitted power of radar network based on a predefined MI threshold.

The MI between the received signal matrix $Y^{q}$ and the target scatterer matrix $G^{q}$ given the knowledge of $S^{q}U^{q}$ is given by:

(8) $$I\left(Y^{q};G^{q}|S^{q}U^{q}\right)=h\left(Y^{q}|S^{q}U^{q}\right)-h\left(Y^{q}|G^{q},S^{q}U^{q}\right)=h\left(Y^{q}|S^{q}U^{q}\right)-h\left(W\right)$$

where h(·) indicates the differential entropy. In order to obtain $I\left(Y^{q};G^{q}|S^{q}U^{q}\right)$, the conditional entropy $h\left(Y^{q}|S^{q}U^{q}\right)$ and the Gaussian white noise entropy h(W) are firstly calculated. The conditional probability density function (PDF) of $Y^{q}$ for a given $S^{q}U^{q}$ is given by:

(9) $$\begin{array}{l} f\left(Y^{q}|S^{q}U^{q}\right)=\prod_{j=1}^{N}f\left(y_{j}^{q}|S^{q}U^{q}\right)\\ =\prod_{j=1}^{N}\frac{1}{\pi^{K}\det\left(\left(S^{q}U^{q}\right)\Sigma_{h_{j}^{q}}{\left(S^{q}U^{q}\right)}^{H}+\sigma_{w}^{2}I_{k}\right)}\times \exp\left\{-{y_{j}^{q}}^{*}{\left[\left(S^{q}U^{q}\right)\Sigma_{h_{j}^{q}}{\left(S^{q}U^{q}\right)}^{H}+\sigma_{w}^{2}I_{k}\right]}^{-1}{y_{j}^{q}}^{T}\right\}\\ =\frac{1}{\pi^{NK}\prod_{j=1}^{N}\det\left(\left(S^{q}U^{q}\right)\Sigma_{h_{j}^{q}}{\left(S^{q}U^{q}\right)}^{H}+\sigma_{w}^{2}I_{k}\right)}\times \exp\left\{-\mathrm{tr}\left[{\left(\left(S^{q}U^{q}\right)\Sigma_{h_{j}^{q}}{\left(S^{q}U^{q}\right)}^{H}+\sigma_{w}^{2}I_{k}\right)}^{-1}Y^{q}{Y^{q}}^{H}\right]\right\} \end{array}$$

where $\Sigma_{h_{j}^{q}}$ denotes the covariance matrix of $h_{j}^{q}$, then the differential entropy of $Y^{q}|S^{q}U^{q}$ can be obtained:

(10) $$\begin{array}{ll} h\left(Y^{q}|S^{q}U^{q}\right) & =-\int f\left(Y^{q}|S^{q}U^{q}\right)\log f\left(Y^{q}|S^{q}U^{q}\right)dY^{q}\\ & =\sum_{j=1}^{N}\log\left[\det\left(\sigma_{w}^{2}I_{K}+\left(S^{q}U^{q}\right)\sum_{h_{j}^{q}}{\left(S^{q}U^{q}\right)}^{H}\right)\right]+NK\log\pi +NK \end{array}$$

Similarly, the PDF of W is given by:

(11) $$f\left(W\right)=\frac{1}{\pi^{NK}\det^{N}\left(\sigma_{w}^{2}I_{K}\right)}\times \exp\left\{-\mathrm{tr}\left[{\left(\sigma_{w}^{2}I_{K}\right)}^{-1}WW^{H}\right]\right\}$$

The differential entropy of W can be obtained:

(12) $$h\left(W\right)=-\int f\left(W\right)\log f\left(W\right)dW=NK\log\sigma_{w}^{2}+NK\log\pi +NK$$

Then the mutual information $I\left(Y^{q};G^{q}|S^{q}U^{q}\right)$ is given by:

(13) $$\begin{array}{ll} I\left(Y^{q};G^{q}|S^{q}U^{q}\right) & =\sum_{j=1}^{N}\log\left[\det\left(\sigma_{w}^{2}I_{K}+\left(S^{q}U^{q}\right)\Sigma_{h_{j}^{q}}{\left(S^{q}U^{q}\right)}^{H}\right)\right]-NK\log\sigma_{w}^{2}\\ & =\sum_{j=1}^{N}\log\left[\det\left(I_{K}+\sigma_{w}^{-2}\left(S^{q}U^{q}\right)\Sigma_{h_{j}^{q}}{\left(S^{q}U^{q}\right)}^{H}\right)\right]\\ & =\sum_{j=1}^{N}\log\left[\det\left(I_{N}+\sigma_{w}^{-2}\Sigma_{h_{j}^{q}}{\left(S^{q}U^{q}\right)}^{H}\left(S^{q}U^{q}\right)\right)\right] \end{array}$$

where Equation (13) follows from:

(14) $$\det\left(I_{r}+AB\right)=\det\left(I_{t}+BA\right)$$

One more assumption is appended:

(5) The transmitted waveforms are orthogonal with different power, then ${s_{i}^{q}}^{T}s_{j}^{q}=0$ (i≠j) can be obtained. Let $E_{i}^{q}={s_{i}^{q}}^{T}s_{i}^{q}$ denotes the transmitted power of radar i when it is assigned to target *q*.

In order to obtain the value of mutual information, a useful lemma should be introduced.

**Lemma** **1.** *Let* A *be an* N×N *positive semi-definite Hermitian matrix with* (i,j)*th entry* $a_{ij}$*. Then the following inequality*

(15) $$\det\left(A\right)\leq \prod_{i=1}^{N}a_{ii}$$

*holds with equality if and only if* A *is diagonal*.

**Proof.**  The proof can be found in reference [30].

The maximum value of $I\left(Y^{q};G^{q}|S^{q}U^{q}\right)$ will be achieved if and only if $I_{N}+\sigma_{w}^{-2}\Sigma_{h_{j}^{q}}{\left(S^{q}U^{q}\right)}^{H}\left(S^{q}U^{q}\right)$ is diagonal based on the lemma. According to Assumptions (1) and (2), it can be determined that the columns of $H^{q}=G^{q}⊙P^{q}$ are no longer identically distributed. The distribution of $h_{j}^{q}$ is $h_{j}^{q}∼CN\left(0,\sigma_{g}^{2}\text{diag}\left({p_{1j}^{q}}^{2},{p_{2j}^{q}}^{2},...,{p_{Nj}^{q}}^{2}\right)\right)$. Therefore the covariance matrix of $h_{j}^{q}$ denoted by $\Sigma_{h_{j}^{q}}=\sigma_{g}^{2}\text{diag}\left({p_{1j}^{q}}^{2},{p_{2j}^{q}}^{2},...,{p_{Nj}^{q}}^{2}\right)$ is a diagonal matrix with positive elements. According to Assumption (5), $S^{qH}S^{q}=\text{diag}\left(E_{1}^{q},E_{2}^{q},...,E_{N}^{q}\right)$ and ${\left(S^{q}U^{q}\right)}^{H}\left(S^{q}U^{q}\right)=\text{diag}\left(u_{1}^{q}E_{1}^{q},u_{2}^{q}E_{2}^{q},...,u_{N}^{q}E_{N}^{q}\right)$ can be obtained.

(16) $$I_{N}+\sigma_{w}^{-2}\Sigma_{h_{j}^{q}}{\left(S^{q}U^{q}\right)}^{H}\left(S^{q}U^{q}\right)=I_{N}+\sigma_{w}^{-2}\sigma_{g}^{2}\text{diag}\left({p_{1j}^{q}}^{2}u_{1}^{q}E_{1}^{q},{p_{2j}^{q}}^{2}u_{2}^{q}E_{2}^{q},...,{p_{Nj}^{q}}^{2}u_{N}^{q}E_{N}^{q}\right)$$

Inserting Equations (16) into (13) and using the lemma, it can be obtained that the true value of $I\left(Y^{q};G^{q}|S^{q}U^{q}\right)$ equal to its maximum value under the assumptions mentioned above.

(17) $$I\left(Y^{q};G^{q}|S^{q}U^{q}\right)=\sum_{i=1}^{N}\sum_{j=1}^{N}\log\left(1+\sigma_{w}^{-2}\sigma_{g}^{2}{p_{ij}^{q}}^{2}u_{i}^{q}E_{i}^{q}\right)$$

## 4. Sensor Selection and Power Allocation Algorithm

Due to the operating principle of the interceptor receiver, the detection probability for radar in an interceptor is related to radar transmitted power. Hence, to achieve the low probability of intercept of radar network, it is necessary to select a suitable radar to track each target and allocate its transmitted power optimally. In this paper, the traceability of each radar in the radar network is defined as η, meaning that each radar can track at most η targets simultaneously. According to Equation (17), it can be found that the MI is related to two variable parameters, including sensor selection index and radar transmitted power. MI is taken as a performance metric, and the main goal of this work is to minimize the total transmitted power of activated radars at each time instant based on a predefined MI threshold. Hence, the optimization problem of sensor selection and power allocation based on LPI at each time instant can be summarized as:

(18) $$\begin{array}{l} \underset{E_{i}^{q},u_{i}^{q}}{\text{min}}\sum_{i=1}^{N}\sum_{q=1}^{Q}u_{i}^{q}E_{i}^{q}\\ s.t.\{\begin{array}{c} \begin{array}{c} \sum_{i=1}^{N}\sum_{j=1}^{N}\log\left(1+\sigma_{w}^{-2}\sigma_{g}^{2}{p_{ij}^{q}}^{2}u_{i}^{q}E_{i}^{q}\right)\geq I_{\text{min}},q\in \left\{1,...,Q\right\}\\ E_{\text{min}}\leq E_{i}^{q}\leq E_{\text{max}},q\in \left\{1,...,Q\right\},i\in \left\{1,...,N\right\}\\ \sum_{i=1}^{N}u_{i}^{q}=1,q\in \left\{1,...,Q\right\}\\ \sum_{q=1}^{Q}u_{i}^{q}\leq \eta ,i\in \left\{1,...,N\right\} \end{array}\\ u_{i}^{q}\in \left\{0,1\right\},q\in \left\{1,...,Q\right\},i\in \left\{1,...,N\right\}\\ h_{j}^{q}∼CN\left(0,\sigma_{g}^{2}\text{diag}\left({p_{1j}^{q}}^{2},{p_{2j}^{q}}^{2},...,{p_{Nj}^{q}}^{2}\right)\right)\\ {\left(S^{q}U^{q}\right)}^{H}\left(S^{q}U^{q}\right)=\text{diag}\left(u_{1}^{q}E_{1}^{q},u_{2}^{q}E_{2}^{q},...,u_{N}^{q}E_{N}^{q}\right) \end{array} \end{array}$$

where $I_{\text{min}}$ is the predefined MI threshold. $\sum_{i=1}^{N}\sum_{j=1}^{N}\log\left(1+\sigma_{w}^{-2}\sigma_{g}^{2}{p_{ij}^{q}}^{2}u_{i}^{q}E_{i}^{q}\right)\geq I_{\text{min}}$ means the MI between the target impulse response and the reflected signal cannot less than a predetermined MI threshold which is based on the necessary MI the radar network required to estimate the targets. The radar transmitted power is constrained by a minimum value $E_{\text{min}}$ and a maximum value $E_{\text{max}}$. $\sum_{i=1}^{N}u_{i}^{q}=1$ means that only a single radar is assigned to each target and $\sum_{q=1}^{Q}u_{i}^{q}\leq \eta$ means that the traceability of each radar is η. The constraint $h_{j}^{q}∼CN\left(0,\sigma_{g}^{2}\text{diag}\left({p_{1j}^{q}}^{2},{p_{2j}^{q}}^{2},...,{p_{Nj}^{q}}^{2}\right)\right)$ and the constraint ${\left(S^{q}U^{q}\right)}^{H}\left(S^{q}U^{q}\right)=\text{diag}\left(u_{1}^{q}E_{1}^{q},u_{2}^{q}E_{2}^{q},...,u_{N}^{q}E_{N}^{q}\right)$ should be satisfied under the assumptions mentioned above.

### 4.1. Power Allocation Optimization Problem Solution

The optimization problem described in Equation (18) is non-convex containing two parameters $u_{i}^{q}$ and $E_{i}^{q}$. For a given $U^{q}$ assuming that radar i is assigned to target *q*, the uniquely sensor selection scheme for target q can be determined. The term $\sum_{i=1}^{N}\sum_{j=1}^{N}\log\left(1+\sigma_{w}^{-2}\sigma_{g}^{2}{p_{ij}^{q}}^{2}u_{i}^{q}E_{i}^{q}\right)$ can be rewritten as $\sum_{j=1}^{N}\log\left(1+\sigma_{w}^{-2}\sigma_{g}^{2}{p_{ij}^{q}}^{2}E_{i}^{q}\right)$ for a given $U^{q}$. The optimization problem described in Equation (18) can be reformulated as an optimization sub-problem with a single parameter $E_{i}^{q}$ for a given sensor selection scheme as follow:

(19) $$\begin{array}{c} \text{min }E_{i}^{q}\\ s.t.\{\begin{array}{c} \sum_{j=1}^{N}\log\left(1+\sigma_{w}^{-2}\sigma_{g}^{2}{p_{ij}^{q}}^{2}E_{i}^{q}\right)\geq I_{\text{min}}\\ E_{\text{min}}\leq E_{i}^{q}\leq E_{\text{max}} \end{array} \end{array}$$

Because the term $\sum_{j=1}^{N}\log\left(1+\sigma_{w}^{-2}\sigma_{g}^{2}{p_{ij}^{q}}^{2}E_{i}^{q}\right)$ is monotonically increasing and concave with respect to $E_{i}^{q}$, Equation (19) can be solved with the bisection method [31] which is a very simple and robust method. The detailed steps of the solution of Equation (19) with the bisection function method can be shown in Algorithm 1.

**Algorithm 1** Bisection Method for Power Allocation**Step (1):** Set $a=E_{\text{min}}$, $b=E_{\text{max}}$, $f\left(E_{i}^{q}\right)=\sum_{j=1}^{N}\log\left(1+\sigma_{w}^{-2}\sigma_{g}^{2}{p_{ij}^{q}}^{2}E_{i}^{q}\right)-I_{\text{min}}$ and the pre-specified accuracy ξ.
**Step (2):** Calculate f(a), if f(a)≥0, return a and stop the algorithm, otherwise, go to Step (3).**Step (3):** Calculate the midpoint of the interval [a,b], $c=\frac{a+b}{2}$.**Step (4):**
If f(c)=0, return c and stop iterating;If f(c)<0, the intercal [a,c] provides no feasible point to satisfy $f\left(E_{i}^{q}\right)\geq 0$, replace (a,f(a)) with (c,f(c));If f(c)>0, the intercal [c,b] provides no feasible point to satisfy $f\left(E_{i}^{q}\right)\geq 0$, replace (b,f(b)) with (c,f(c)).**Step (5):** If convergence is satisfactory (that is, |a−b|<ξ), then return a or b, and stop iterating, otherwise, go to Step (3).

### 4.2. Sensor Selection Optimization Problem Solution

According to the discussions in the last subsection, the minimum radar transmitted power of radars for tracking each target can be obtained. By solving the N×Q times optimization problem of Equation (19), the minimum transmitted power of each radar for all possible schemes can be obtained. Define the minimum transmitted power matrix $E_{\mathrm{opt}}$ with elements $E_{i,\mathrm{opt}}^{q}$ similar to Table 1.

Sensor selection matrix $U_{\mathrm{opt}}$ with elements $U_{i}^{q}$ is shown in Table 2.

The optimization sub-problem of sensor selection at each time instant can be posed as:

(20) $$\begin{array}{l} \underset{E_{i,opt}^{q},u_{i}^{q}}{\text{min}}\sum_{i=1}^{N}\sum_{q=1}^{Q}u_{i}^{q}E_{i,opt}^{q}\\ s.t.\{\begin{array}{c} \begin{array}{c} \sum_{i=1}^{N}u_{i}^{q}=1\\ \sum_{q=1}^{Q}u_{i}^{q}\leq \eta \end{array}\\ u_{i}^{q}\in \left\{0,1\right\} \end{array} \end{array}$$

Equation (20) can be solved by exhaustive search with exponential complexity of $O\left(N^{Q}\right)$. For reducing the complexity, a sensor selection algorithm with lower complexity is proposed as shown in Algorithm 2. 

**Algorithm 2** Sensor Selection Algorithm with Lower Complexity**Step (1):** Form the required minimum transmitted power matrix $E_{\mathrm{opt}}\in {\mathbb{C}}^{N\times Q}$ according to Section 4.1.**Step (2):** Find the minimum power of each column, radar in relation to the minimum power is assigned to the target of this column.**Step (3):** Choose a target priority order for the sensor selection. According to the target priority order from low to high, if the assignment of target has inconsistencied with condition $\sum_{q=1}^{Q}u_{i}^{q}\leq \eta$, shift to the next minimum power by holding others.**Step (4):** Find the best sensor selection scheme which satisfy condition $\sum_{i=1}^{N}u_{i}^{q}=1$ and condition $\sum_{q=1}^{Q}u_{i}^{q}\leq \eta$, and then calculate the value of $\sum_{i=1}^{N}\sum_{q=1}^{Q}u_{i}^{q}E_{i,\mathrm{opt}}^{q}$.**Step (5):** Choose another target priority order and return to Step (3) until all permutation of target priority order has been investigated.**Step (6):** Sensor selction matrix can be formed with the minimum $\sum_{i=1}^{N}\sum_{q=1}^{Q}u_{i}^{q}E_{i,\mathrm{opt}}^{q}$ of all target priority orders.

### 4.3. Target State Estimation

In this paper, a centralized tracking method is adopted to estimate the target state. The measurements from all receivers are sent to the fusion center through communication links with negligible time-synchronization errors. The KNN algorithm is adopted for data association between observed data and targets. The KNN algorithm can be used in a variety of applications, including knowledge discovery, data mining and multimedia databases. In this paper, it works based on Euclidean distance [32] from the observed data to the labeled data of each target so as to determine the k-nearest neighbors of the observed data. After the k-nearest neighbors are gathered, the majority of these k-nearest neighbors can determine “which target the observed data belongs to”.

Let $x_{k}^{q}={\left[x_{k}^{q},y_{k}^{q},{\dot{x}}_{k}^{q},{\dot{y}}_{k}^{q}\right]}^{T}$ denote the state vector of target q at time instant k, with $\left[x_{k}^{q},y_{k}^{q}\right]$ denoting the position of target q and $\left[{\dot{x}}_{k}^{q},{\dot{y}}_{k}^{q}\right]$ denoting the velocity of target q. Target motion is expressed by the state equation as:

(21) $$x_{k+1}^{q}=Fx_{k}^{q}+w_{k}^{q}$$

where F is the transition matrix and $w_{k}^{q}$ is the process noise. The measurement model for centralized target tracking is given by:

(22) $$z_{k}^{q}={\left[z_{k}^{q1},z_{k}^{q2},...,z_{k}^{qN}\right]}^{T}+n_{k}^{q}={\left[h_{d}^{q1},h_{q}^{q1},h_{d}^{q2},h_{\theta }^{q2},...,h_{d}^{qN},h_{\theta }^{qN}\right]}^{T}+n_{k}^{q}$$

where $h_{d}^{qi}=\sqrt{{\left(x_{k}^{q}-x^{i}\right)}^{2}+{\left(y_{k}^{q}-y^{i}\right)}^{2}}$, $h_{\theta }^{qi}=\arctan\left(\frac{y_{k}^{q}-y^{i}}{x_{k}^{q}-x^{i}}\right)$, i=1,2,...,N. $\left(x^{i},y^{i}\right)$ is the position of radar i, $n_{k}^{q}$ is the measurement noise. For simplicity, $w_{k}^{q}$ and $n_{k}^{q}$ are assumed to be Gaussian white noise with zero mean.

As shown in reference [24], SIR-PF is a non-linear and non-Gaussion filter which directly approximates the PDF using finite particles. Since the measurement model of target tracking in this paper is non-linear, SIR-PF technique can be employed at the fusion center to obtain the state estimation of each target.

Overall, Algorithm 3 presents the detailed steps of target state estimation with sensor selection and power allocation.

**Algorithm 3** General Steps of Target Tracking**Initialization:** Let k=1, set $U_{k-1,\mathrm{opt}}=U_{0}$, $E_{k-1,\mathrm{opt}}=E_{0}$, assume an initial PDF $p\left(x_{0}^{q}\right)$ and the particle number L.

**Iteration:** For k=1,2...**Step (1):** Data association: Calculate the Euclidean distance between observed data and labeled data of each target, and then which target the observed data belong to is determined by the majority of its k-nearest neighbors;**Step (2):** Draw L samples according to $p\left(x_{k}^{q}|x_{k-1}^{q}\right)$ and obtain $x_{k}^{q\left(l\right)}$;**Step (3):** Measurement update: For l=1,...,L, give the measurement vector $z_{k}^{q}\left(U_{k}^{q},E_{k}^{q}\right)$, and define the weight $w_{k}^{q\left(l\right)}=p\left(z_{k}^{q}\left(U_{k}^{q},E_{k}^{q}\right)|x_{k}^{q\left(l\right)}\right)$;**Step (4):** Normalizing: $w_{k}^{q\left(l\right)}=w_{k}^{q\left(l\right)}/\sum_{l=1}^{L}w_{k}^{q\left(l\right)}$;**Step (5):** Resampling: Take L samples with replacement from the set ${\left\{x_{k}^{q\left(l\right)}\right\}}_{l=1}^{L}$, where the probability to take sample l is $w_{k}^{q\left(l\right)}$ and let $w_{k}^{q\left(l\right)}=1/L$;**Step (6):** Calculate $U_{k,\mathrm{opt}}$ and $E_{k,\mathrm{opt}}$ according to Equation (18);**Step (7):** Send $U_{k,\mathrm{opt}}$ and $E_{k,\mathrm{opt}}$ to all radars.

## 5. Numerical Simulations

In this section, the LPI performance of the radar network based on the sensor selection and power allocation algorithm proposed in this paper is evaluated. In the simulations, we assume the radar network is composed of four monostatic radars. All radars in the network system have the same parameters, which are shown in Table 3.

In order to evaluate the effect of radar deployment, two different geometrical arrangements of radar nodes which constitute the network system are chosen for this analysis. In the first case, four monostatic radars are deployed as a square, while in the second case, the same number radars are positioned in a straight line.

**Figure 1:**
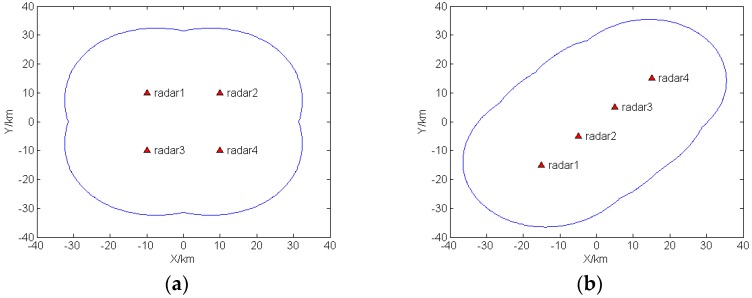
Two-dimensional coverage plot: (**a**) Case 1; (**b**) Case 2.

In order to evaluate the sensitivity properties of radar network, the SNR threshold has been set as 13 dB. The two-dimensional coverage plot of the first case is shown in Figure 1a, and the coverage plot of the second case is shown in Figure 1b. Targets located outside the boundary cannot be detected.

The predefined MI threshold mentioned in the optimal condition of Equation (18) can be calculated with the condition that distance between the target and the activated radar is equal to the minimum range between boundary point and the activated radar, and the transmitted power is equal to the maximum peak power. For simplicity, set $\sigma_{w}=1$, $\sigma_{g}=1$, $\kappa =10^{11}$. For the first case, the minimum range between boundary point and the transmitted radar is 21.6175 km, $I_{\text{min}1}=18.5321$, while in the second case, the minimum range between boundary point and the transmitted radar is 21.0999 km, $I_{\text{min}2}=17.9801$.

**Figure 2:**
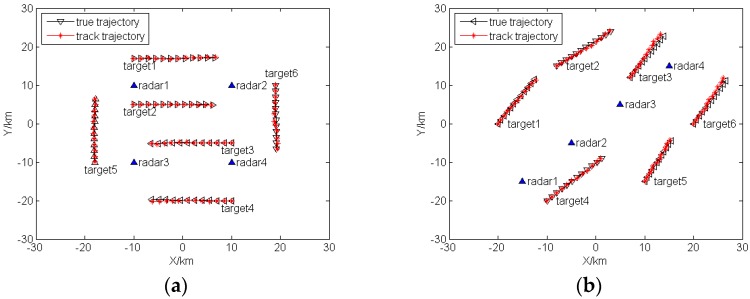
True target trajectories and track trajectories: (**a**) Case 1; (**b**) Case 2.

Assuming the target number Q=6, target trajectories are inside the boundary of coverage plot. The sampling interval is set as 2 s, and the total tracking time is 22 s. The true target trajectories and track trajectories are shown in Figure 2.

**Figure 3:**
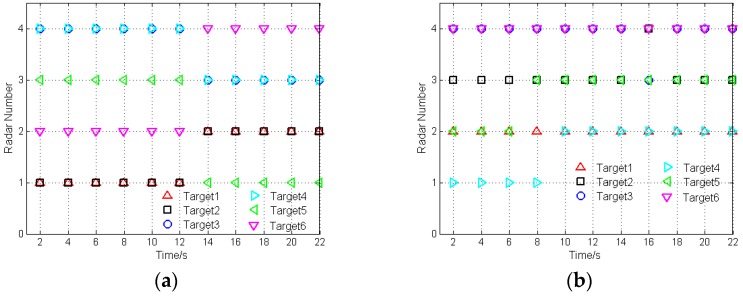
Sensor selection results: (**a**) Case 1; (**b**) Case 2.

To obtain the minimum transmitted power matrix shown in Table 1, a 24-time bisection method is employed to solve Equation (19) at each sampling instant for each case. Then, the sensor selection algorithm with lower complexity proposed in Section 4.2 will be applied to solve Equation (20) at each sampling instant with the minimum transmitted power results. It can be assumed that the traceability of each radar in the radar network at each time instant is η=2. Additionally, the sensor selection results of case 1 and case 2 are displayed in Figure 3.

**Figure 4:**
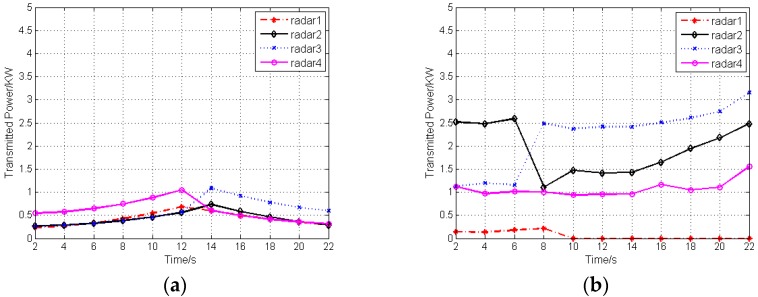
Transmitted power of radars after sensor selection and power allocation: (**a**) Case 1; (**b**) Case 2.

Take case 1 as an example. In the initial stage, target 1 and target 2 are the closest to radar 1; target 3 and target 4 are the closest to radar 4; target 5 is the closest to radar 3; target 6 is the closest to radar 2. As shown in Figure 3a, the radars are assigned to the closest targets. During the optimization process of sensor selection and power allocation, with the target movement, the radar, which is the closest to the target, is selected to operate in an active way to track this target, if the condition of η=2 is satisfied. Figure 4 depicts the transmitted power of radars after sensor selection and power allocation during the target tracking process.

It can be seen that a significant reduction in the transmitted power of radars will be achieved by adopting the proposed algorithm. In other words, the LPI performance of the radar network can be enhanced after sensor selection and power allocation. 

**Figure 5:**
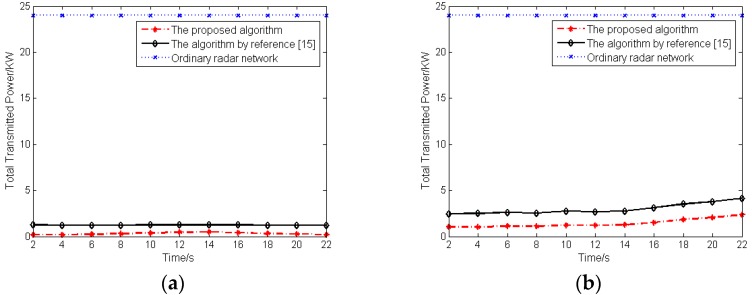
Total transmitted power comparison: (**a**) Case 1; (**b**) Case 2.

In order to compare the effect of the proposed algorithm with the other algorithms on the LPI performance of radar network, Figure 5 illustrates the comparison of the total transmitted power of radar network with the same geometrical arrangement and radar parameters when just tracking target 1 by employing the proposed algorithm of this paper, the algorithm of reference [15] and an ordinary radar network. The algorithm proposed in reference [15] is valuable to improve the LPI performance of the radar network by allocating the transmitted power of radars for single target tracking without sensor selection. All radars of an ordinary radar network with equal power allocation have a constant transmitted power of 6 KW.

As shown by the results, the total transmitted power of the radar network, which employs the proposed algorithm of this paper, is smaller than reference [15] and strictly smaller than an ordinary radar network. Furthermore, the results provided in Figure 5 show that the best LPI performance of the radar network can be obtained by employing the algorithm proposed in this paper.

## 6. Conclusions

In this paper, the problem of LPI design in radar network architecture for multiple-target tracking has been investigated, where an LPI optimization framework based on sensor selection and power allocation under a predefined MI threshold has been proposed. The bisection method was employed to tackle the sub-problem of power allocation for each sensor selection scheme. The sub-problem of sensor selection is solved by a lower-complexity algorithm based on the allocated powers. Simulations demonstrate that a significant reduction of total transmitted power from the radar network can be achieved through the proposed sensor selection and power allocation algorithm, compared with an ordinary radar network and the algorithm of reference [15]. Hence, the LPI performance of a radar network for multiple-target tracking can be effectively improved.

## Figures and Tables

**Table 1:** Minimum transmitted power matrix.

Minimum Transmitted Power		Targets			
		$T_{1}$	$T_{2}$	...	$T_{Q}$
Radars	$R_{1}$	$E_{1,\mathrm{opt}}^{1}$	$E_{1,\mathrm{opt}}^{2}$	…	$E_{1,\mathrm{opt}}^{Q}$
	$R_{2}$	$E_{2,\mathrm{opt}}^{1}$	$E_{2,\mathrm{opt}}^{2}$	…	$E_{2,\mathrm{opt}}^{Q}$
	...	...	...		...
	$R_{N}$	$E_{N,\mathrm{opt}}^{1}$	$E_{N,\mathrm{opt}}^{2}$	…	$E_{N,\mathrm{opt}}^{Q}$

**Table 2:** Sensor selection matrix.

Sensor Selection Index		Targets			
		$T_{1}$	$T_{2}$	...	$T_{Q}$
Radars	$R_{1}$	$u_{1}^{1}$	$u_{1}^{2}$	…	$u_{1}^{Q}$
	$R_{2}$	$u_{2}^{1}$	$u_{2}^{2}$	…	$u_{2}^{Q}$
	...	...	...		...
	$R_{N}$	$u_{N}^{1}$	$u_{N}^{2}$	…	$u_{N}^{Q}$

**Table 3:** Radar parameters.

Single Radar Maximum Peak Power	Radar Transmitted Antenna Gain	Radar Received Antenna Gain	Radar Frequency	Radar Band Width	Radar System Loss
6 KW	30 dB	30 dB	3 GHz	1 MHz	5 dB
